# The association between interleukin-19 concentration and diabetic nephropathy

**DOI:** 10.1186/s12882-017-0488-7

**Published:** 2017-02-15

**Authors:** Li Li, Xu-gan Jiang, Juan-Yu Hu, ZHeng-Qing Yu, Jian-Yong Xu, Fan Liu, Guang-Chun Zhao, Lei Zhang, Hui-Ming Gu, Si-Jing Zhang, Jin Meng

**Affiliations:** 1 Department of Clinical Laboratory, Binhai County Hospital, Binhai, Jiangsu Province China; 2 School of Medicine, Jiangsu University, Zhenjiang, China; 3 Department of Clinical Laboratory, Binhai County Second Hospital, Binhai, Jiangsu Province China

**Keywords:** Interleukin-19, Diabetic nephropathy, Albuminuria, High-sensitivity C-reactive protein

## Abstract

**Background:**

Interleukin-19 (IL-19) is a newly discovered cytokine belonging to the Interleukin-10(IL-10) family. IL-19 have indispensable functions in many inflammatory processes and also can induce the angiogenic potential of endothelial cells. The purpose of present study was to investigate the relation of serum interleukin-19 (IL-19) levels with diabetic nephropathy (DN).

**Methods:**

Two hundred study groups of patients with type 2 diabetes mellitus (T2DM) (109 males and 91 females) were recruited, included normoalbuminuria(*n* = 102), microalbuminuria(*n* = 72) and macroalbuminuria(*n* = 26) . The 50 healthy blood donors were enrolled for the control group. All subjects were assessed for: IL-19, High-sensitivity C-reactive protein (Hs-CRP), Cystatin C, urinary albumin excretion rate (UAE) and glycosylated hemoglobin A1c(HbA1c).

**Results:**

The serum IL-19 levels in DN patients were found to be significantly higher compared to controls. IL-19 levels were significantly positively correlated with Hs-CRP, Cystatin C, UAE and HbA1c(*r* = 0.623, 0.611,0.591 and 0.526 respectively, *P* < 0.01). Multivariable logistic regression analysis showed IL-19 levels (*P* = 0.01) were found to be independently associated with patients with DN.

**Conclusions:**

IL-19 is significantly positive correlated with UAE and Cystatin C. IL-19 may play an important role that contributes to the progression of diabetic nephropathy.

## Background

Type 2 diabetes mellitus (T2DM) is a metabolic disease. Hyperglycemia is the apparent feature of T2DM due to the deficiency in peripheral insulin effects (insulin resistance). The development of both macrovascular and microvascular complications is a primary cause of morbidity and mortality in diabetes that rapidly leads to premature death [1, 2]. Vascular complications are considered for the dominant causeof the life span and the quality of life in T2DM, and it is important to understand the risk factors in order to prevent the development and progression of the complications [3]. Diabetic nephropathy (DN) is a major microvascular complication of diabetes mellitus(DM), it is the leading cause of end-stage renal disease. Inflammation plays some important roles in the pathogenesis of DN. Leukocytes, macrophages and monocytes all involve in the process of DN [4, 5], and proinflammatory cytokines and inflammatory markers are strongly associated with the development of DN [6, 7].

High-sensitivity C-reactive protein (Hs-CRP), which is a marker of inflammation, has been reported to be associated with development of DN [8]. Albuminuria is one of the first asymptomatic clinical features of microvascular damage in DM. It has been shown that microalbuminuria and macroalbuminuria are associated with progressive renal function loss [9]. Hs-CRP levels may predict the development of albuminuria in some studies in type 1 and type 2 DM patients [8, 10].

Interleukin-19 (IL-19) is a newly discovered cytokine within the Interleukin-10(IL-10) family. This protein can stimulate the production of IL-10 from human peripheral blood mononuclear cells [11, 12]. It has been reported that IL-19 can promote the T-helper2 (Th2) response, which is associated with a wide variety of allergic (i.e., asthma and atopic dermatitis [13–15], type 1 diabetes [16], and cardiovascular disease [17, 18]. IL-19 have indispensable functions in many inflammatory processes and also can induce the angiogenic potential of endothelial cells [19, 20].

Several recent studies have revealed that the roles of IL-19 in development of vascular inflammatory diseases such as atherosclerosis, restenosis, and coronary artery transplant vasculopathy. Our previous also reported that IL-19 is closely related to T2DM with vascular complications [21]. However, whether there are some association between IL-19 concentration and DN have not been revealed clearly yet. The aim of this study was to determine the concentrations of IL-19 in DN and to investigate the relation of IL-19 with microalbuminuria/macroalbuminuria in DN.

## Methods

### Patients selection

200 patients with type 2 diabetes mellitus (109 males and 91 females, age 60 ± 10.3 years) from July to December in 2015 were admitted to this study. According to urinary albumin excretion rate (UAE), the patients were divided into three groups:T2DM with normoalbuminuria (*n* = 102, UAE < 30 mg/24 h), T2DM with microalbuminuria (*n* = 72, UAE: 30–300 mg/24 h) and T2DM with macroalbuminuria (*n* = 26, UAE ≥300 mg/24 h). The exclusion criteria included patients with type 1 diabetes and those previously diagnosed with urolithiasis, patients with confounding factors for proteinuria, recent or current viral hepatitis or cirrhosis of liver, medical history of clinical cardiovascular disease, chronic lung disease, or acute or chronic infections. Fifty healthy individuals matched for age and sex with the patients were included in the study as a control group (26 males and 24 females, age 58 ± 11 years). The study was approved by the Human Investigation Committee of BinHai County hospital, and written informed consent was obtained from all the study participants. The study was carried out in accordance with the guidelines of the Declaration of Helsinki.

### Laboratory analysis

Venous blood samples were obtained from each participant upon hospital admission. All samples were collected in vacuum blood collection tubes with a clot activator, which blood was centrifuged at 1000 × *g* and 4 °C for 10 min. Serum was separated and aliquoted then stored at −70 °C until analysis.

IL-19 was determined by the enzyme-linked immunosorbent assay (ELISA) kits((R&D Systems, Minneapolis, MN, USA). Urinary albumin and Hs-CRP concentrations were assessed using the particle enhanced immuoturbidimetric method(BNProspec, SIEMENS,Germany). HbA1c was measured by liquid chromatography (G8-90SL, Tosoh, Japan). Fasting plasma glucose(FPG), total cholesterol(TC), high density lipoprotein(HDL-C) and LDL cholesterol(LDL-C) were measured by enzymatically.

### Statistical analysis

The results was expressed as mean ± S.D. Comparisons between two groups were performed using Student’s t-test upon test of normality and equality of variances. Spearman’s or Pearson’s method correlation analysis was carried out to determine the association of each group. *P* < 0.05 was considered statistical significance. All analyses were performed using SPSS 17.0 (SPSS, Inc., Chicago, IL, USA).

## Results

### Clinical characteristics of participants

As expected, Hs-CRP, HbA1c, UAE, Cystatin C, TC, and triglyceride concentrations were significantly higher and HDL-C was lower in T2DM patients compared to the controls. Serum IL-19 concentration was significantly higher in T2DM patients than in controls, there were differences between T2DM subgroups (Table 1).

**Table 1:** Clinical characteristics and research indexes between the different studied groups

Characteristics	Controls	T2DM		T2DM with		*P*-value
			Normoalbuminuria	Microalbuminuria	Macroalbuminuria	
n	50	200	102	72	26	–
Age (years) Sex (males/females)	58 ± 11 26/24	60 ± 10.3 109/91	61 ± 10 58/44	59 ± 9.8 39/33	60 ± 11 12/14	0.25 –
Duration of diabetes (years) BMI (kg/m2)	– 25 ± 5	9 (4–13) 28 ± 2	8 (5–11) 23 ± 1.5	10 (6–13) 28 ± 1.6	10 (7–15) 32 ± 3.0	0.210 0.001
SBP (mm Hg)	127 ± 15	130 ± 16	132 ± 15	135 ± 16	138 ± 17	0.025
DBP (mm Hg)	75 ± 7	83 ± 6	81 ± 4	82 ± 5	84 ± 5	0.037
TC (mmol/L)	4.6 ± 1.0	5.7 ± 1.6	5.6 ± 1.8	5.7 ± 1.4	5.9 ± 1.5	0.825
Triglycerides (mmol/L)	1.4 ± 0.6	2.6 ± 1.5	2.1 ± 1.3	2.4 ± 1.5	2.6 ± 1.6	0.874
HDL-C (mmol/L) LDL-C (mmol/L)	1.5 ± 0.5 3.1 ± 0.9	1.3 ± 0.5 2.5 ± 0.7	1.2 ± 0.4 2.6 ± 0.8	1.3 ± 0.4 2.3 ± 0.6	1.1 ± 0.4 2.6 ± 1.0	<0.001 0.058
IL-19 (pg/ml) Cystatin C (mg/L)	13.2. ± 9.5 0.6 ± 0.2	40.5 ± 12.0 1.12 ± 0.7	35.8 ± 12.3 1.32 ± 0.5	49.4 ± 13.6 2.05 ± 1. 0	61.3 ± 18.2 3.87 ± 1.1	<0.001 0.03
UAE (mg/24 h) Hs-CRP (mg/L)	7 ± 2 0.7 ± 0.3	140 ± 120 3.6 ± 1.5	18 ± 14 3.0 ± 0.9	180 ± 130 4.3 ± 1.2	510 ± 225 5.6 ± 1.0	0.001 <0.001
HbA1c (%)	5.4 ± 0.5	8.5 ± 1.8	8.3 ± 1.5	8.7 ± 2.0	8.6 ± 1.8	<0.001

Data are presented as mean ± SD, *BMI* body mass index, *SBP* systolic blood pressure, *DBP* diastolic blood pressure, *TC* total cholesterol, *HDL-C* high-density lipoprotein cholesterol, *LDL-C* low-density lipoprotein cholesterol, *IL-19*, interleukin-19, *UAE* urinary albumin excretion, *HS-CRP* high-sensitivity C-reactive protein

### IL-19 levels in T2DM with normoalbuminuria, microalbuminuria and macroalbuminuria

**Fig. 1:**
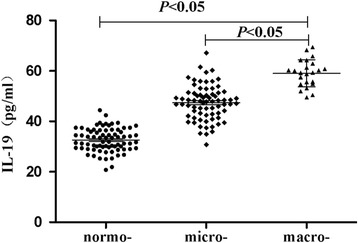
IL-19 levels in T2DM patients with normo-, micro- and macroalbuminuric groups

In 200 T2DM patients, 72 patients had microalbuminuria and 23 patients had macroalbuminuria. There was a pronounced increase in IL-19 of the macroalbuminuric group when compared to the microalbuminuric and normoalbuminuric group. We observed that there was significant difference in concentrations of IL-19 between T2DM patients with normo-, micro- and macroalbuminuric groups (*P* < 0.05) (Fig. 1).

### Correlation analysis

**Fig. 2:**
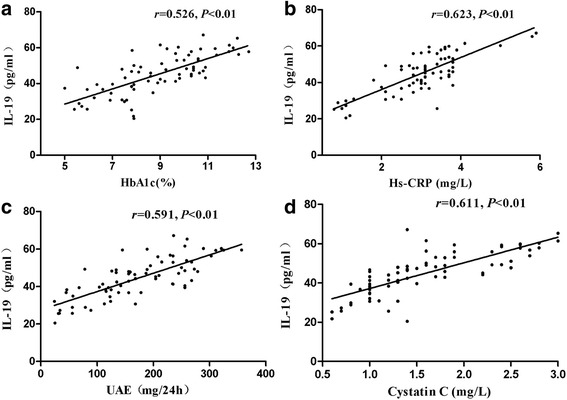
Serum IL-19 levels were positively correlated with HbA1c (**a**), Hs-CRP (**b**), UAE (**c**), CystatinC (**d**) respectively

Serum IL-19 levels showed positively correlation with HbA1c and Hs-CRP respectively (*r* = 0.526 and 0.623 respectively, *P* < 0.01). There was also a strong correlation between IL-19 and UAE,Cystatin C (*r* = 0.591 and 0.611 respectively, *P* < 0.01) (Fig. 2).

### Multivariable logistic regression analyses

In multivariable logistic regression analysis, IL-19 levels (95% CI, 20.1 to 62.9, *P* = 0.01) alone showed a significant positive association with DN even after adjusting for age, gender, hypertension, and blood fat.

## Discussion

Diabetic nephropathy is a syndrome characterized by a progressive increase in the excretion of urinary albumin, elevated blood pressure coupled with glomerular lesions leading ultimately to loss of glomerular filtration and eventually end stage renal failure [22]. Both type 1 diabetes and type 2 diabetes are associated with increased risk of macro- and micro-vascular complications. Apart from the traditional metabolic and hemodynamic risk factors, chronic inflammation is increasingly being regarded as a major risk factor for DN [23, 24].

Proinflammatory cytokines play an important role in the establishment of arteriolosclerosis [25, 26] and kidney injury [27, 28]. Inflammatory cytokines are involved in the development of microvascular diabetic complications, including diabetic nephropathy [29]. IL-19 is a recently described IL-10 family member and the amino acid identity between IL-19 and IL-10 is 20%, but IL-19 does not share the same receptor with IL-10. From human monocytes, B and T lymphocytes, IL-19 can be detected, and the expression of IL-19 can be upregulated in these cells by inflammatory stimuli [30, 31]. It is reported that IL-19 expression is inhibited in immune cells, and our knowledge of the function of this cytokine is from experiments performed in inflammatory cells and which play some indispensable functions in many inflammatory processes [32]. Cuneo et al. reported that inflammatory cytokines and inflammatory stimuli can prompted IL-19 to express, the expression of IL-19 is ascribed in injured and stimulated vascular smooth muscle cells [33]. Our findings showed that IL-19 concentration is elevated in patients with T2DM and IL-19 concentration is significantly higher in macroalbuminuric and microalbuminuric patients than normoalbuminuric patients. Multivariable logistic regression analysis showed IL-19 levels were independently associated with DN. These results suggest that IL-19 involved in the inflammatory reaction and play a significant role in the progression of DN.

Chronic endothelial inflammation is a major risk factor in the occurring of diabetic complications and has a pathogenic role in the progression of DN [34]. High-sensitivity C-reactive protein, which as a marker of inflammation has been reported to be associated with the risk of DM complications [35–37]. Yamaoka-Tojo et al. [38] showed that CRP may deteriorate the inflammatory cascade in tissue injury in addition to initiating endothelial damage and atherosclerosis. In agreement with the previous reports, this study also showed that Hs-CRP were significantly elevated in the DN group in comparing with the control group. We also found a positive correlation between plasma concentrations of IL-19 and Hs-CRP. It is well known that IL-19 is expressed in human endothelium cell can be detected in monocytes and macrophages infiltrate the glomeruli and /or interstitium in the kidney tissue in patients with DM. So, infiltrating macrophages may be responsible for increased levels of IL-19 and IL-19 contributes to the inflammatory response. These data further indicate that IL-19 and inflammation reaction are closely concerned with the progression of nephropathy.

Jennings et al. [39] reported that IL-19 significantly correlated with estimated glomerular filtration rate levels. In this study, IL-19 positively correlated with cystatin C, which is a marker of renal function, and UAE, which is a marker of renal injury respectively. Possible explanations for our finding are an association of IL-19 with proteinuria in DN. First, elevated levels of IL-19 may be the result of pre-existing atherosclerosis in T2DM patients with microalbuminuria. Second, elevations of CRP and IL-19 may directly alter glomerular function and thus be causally involved in the development of albuminuria. Third, there is a potential link between IL-19 and glomerular function. IL-19 may influence the metabolism of the vascular endothelium and the glomerular basement membrane and are involved in the etiology of microalbuminuria.

In the current study, HbA1c in the microalbuminuric/macroalbuminuric diabetic group were significantly increased compared to normoalbuminuric and control groups. This study is in agreement with the previous studies, which have suggested that hyperglycemia is the driving force for the development of DN.We also showed a positive correlation between IL-19 concentration and HbA1c. Our results suggest that long-term hyperglycemia may increase the expression of IL-19 via stimulating endothelial cells,which result in local inflammation and accelerate endothelial damage and atherosclerosis.

The limitations of this study should be noted. This cross-sectional study population is relatively small. In addition, the present clinical study provides evidence that the correlation between IL-19 and the progression of DN, but the cause–effect relationship in DN is not addressed. Therefore, the mechanism requires further investigation.

## Conclusions

The results of present study show that IL-19 levels was significantly elevated in the patients with diabetic nephropathy and was associated with Hs-CRP, Cystatin C, UAE and HbA1c. The results suggest that IL-19 has an important role in the acceleration of glomerular injury in addition to its inflammatory effect on this pathophysiology, and provide further insights into the understanding of IL-19 as the possible effectiveness of anti-inflammatory therapy for DN treatment and prevention.

## Abbreviations

BMI: Body mass index
DBP: Diastolic blood pressure
DN: Diabetic nephropathy
ELISA: Enzyme-linked immunosorbent assay
FPG: Fasting plasma glucose
HbA1c: Glycosylated hemoglobin A1c
HDL-C: High-density lipoprotein cholesterol
HS-CRP: High-sensitivity C-reactive protein
IL-10: Interleukin-10
IL-19: Interleukin-19
LDL-C: Low-density lipoprotein cholesterol
SBP: Systolic blood pressure
T2DM: Type 2 diabetes mellitus
TC: Total cholesterol
Th2: T-helper2
UAE: Urinary albumin excretion
